# Understanding Individual Differences in Happiness Sources and Implications for Health Technology Design: Exploratory Analysis of an Open Dataset

**DOI:** 10.2196/65658

**Published:** 2025-01-29

**Authors:** Edel Ennis, Raymond Bond, Maurice Mulvenna, Colm Sweeney

**Affiliations:** 1 School of Psychology Ulster University Coleraine United Kingdom; 2 School of Computing Ulster University Belfast United Kingdom

**Keywords:** happiness, sexes, age, marital status, parents, affections, achievements, datasets, digital health, well-being, mental health, digital mental health interventions, regression analyses, evidence based

## Abstract

**Background:**

Psychologists have developed frameworks to understand many constructs, which have subsequently informed the design of digital mental health interventions (DMHIs) aimed at improving mental health outcomes. The science of happiness is one such domain that holds significant applied importance due to its links to well-being and evidence that happiness can be cultivated through interventions. However, as with many constructs, the unique ways in which individuals experience happiness present major challenges for designing personalized DMHIs.

**Objective:**

This paper aims to (1) present an analysis of how sex may interact with age, marital status, and parental status to predict individual differences in sources of happiness, and (2) to present a preliminary discussion of how open datasets may contribute to the process of designing health-related technology innovations.

**Methods:**

The HappyDB is an open database of 100,535 statements of what people consider to have made them happy, with some people asking to consider the past 24 hours (49,831 statements) and some considering the last 3 months (50,704 statements). Demographic information is also provided. Binary logistic regression analyses are used to determine whether various groups differed in their likelihood of selecting or not selecting a category as a source of their happiness.

**Results:**

Sex and age interacted to influence what was selected as sources of happiness, with patterns being less consistent among female individuals in comparison with male individuals. For marital status, differences in sources of happiness were predominantly between married individuals and those who are divorced or separated, but these were the same for both sexes. Married, single, and widowed individuals were all largely similar in their likelihood of selecting each of the categories as a source of their happiness. However, there were some anomalies, and sex appeared to be important in these anomalies. Sex and parental status also interacted to influence what was selected as sources of happiness.

**Conclusions:**

Sex interacts with age, marital status, and parental status in the likelihood of reporting affection, bonding, leisure, achievement, or enjoying the moment as sources of happiness. The contribution of an open dataset to understanding individual differences in sources of happiness is discussed in terms of its potential role in addressing the challenges of designing DMHIs that are ethical, responsible, evidence based, acceptable, engaging, inclusive, and effective for users. The discussion considers how the content design of DMHIs in general may benefit from exploring new methods informed by diverse data sources. It is proposed that examining the extent to which insights from nondigital settings can inform requirements gathering for DMHIs is warranted.

## Introduction

### Exploring Individual Differences in Sources of Happiness, and the Role of Open Datasets in Health Technology Design

Well-being is founded upon positive emotions, engagement, relationships, meaning, and accomplishments [1]. Different people will derive well-being from each of these 5 building blocks to varying degrees [1]. The premise is that individuals need to focus on positive emotions, engage with life and the activities around them, strive for meaningful relationships and social connections, try to find meaning in life, and have goals and ambitions. Later discussions of the model have concluded life is easier and more enjoyable when our health is optimal. Much literature uses terms such as “happiness,” “subjective well-being,” “thriving,” and “flourishing” interchangeably [2,3]. There is no complete consensus on the relationship between happiness and well-being, but happiness is at least considered to be a component of well-being.

At the very basic level, happiness is an example of a positive emotion that contributes to well-being, with joy, love, and gratitude representing additional examples [4]. The links between happiness and well-being have led to great interest in the science of happiness. This investigates what constitutes happiness; what makes people happy; and what we can do, to ourselves or others, to feel happier [5]. While genetics and life circumstances (eg, finances, job, and material belongings) play roles, 40% of our happiness is determined by behaviors that are under our control [6].

Open data represent a crucial element of the open science movement [7]. These datasets are publicly available, without any restrictions on their use or distribution [7]. The growth of the open science movement has led to an increased availability of open datasets and consideration of how we can reap maximum benefits from these [7]. Example benefits have included transparency and the capacity to amalgamate large datasets, which can give comprehensive insights to inform data-driven decision-making, for example, informing targeted interventions or data-driven resource allocation [7]. Open datasets also allow analysis from a variety of diverse perspectives and thus represent a powerful resource for developing insights into the underlying meaning of psychological constructs [8]. This in turn facilitates enhanced precision and clarity within theories and definitions, an understanding of the meaning of the construct across different cultures and contexts, the development of improved means of measurement, and the development of more effective interventions and treatment options [8].

The HappyDB is an example of a data source that has advanced our understanding of the individual meaning and sources of happiness. The HappyDB is a dataset containing over 100,000 happy statements [9] that is freely available on the web [10]. Some people were asked to consider what had brought them happiness in the past 24 hours, and some were asked to consider the last 3 months. Individuals could make more than 1 entry, but not more than 3 entries. Happiness does not always mean the same thing. Short-term happiness (24 hours) tends to be associated with more daily tasks such as “Exercise,” “Nature,” and “Leisure,” whereas longer-term happiness is more likely to come from loved ones or achievements [9]. Sources of happiness vary cross-culturally [11].

Based on studying happiness within a Latin-American sample, Pena-Lopez et al [12] stressed the need to understand the individual determinants of happiness. Focusing on the words used to express happiness within the HappyDB as opposed to the categories of the sources of happiness, Mohamed and Mostafa [13] used logistic regression, gradient boosting, and the fastText deep learning algorithm, and they found that demographics did influence the meaning and sources of happiness. For example, male individuals expressed greater interest in games and gadgets as sources of happiness, while female individuals discussed family and friends to a greater extent [13]. Parents placed more discussion around the well-being of their families and children, whereas nonparents placed their emphasis on friends, games, eating out, pets, and watching television [13]. Unmarried people focused mostly on dating, friendship, food, and exercise as sources of happiness, whereas married people focused mainly on children and family [13]. Age brackets also differed in how they expressed happiness [13]. Other research studies have also shown differences between demographic groups in the factors associated with happiness levels [14,15].

As noted earlier, sex influences the sources of happiness. Age, marital status, and parenthood status also influence the sources of happiness. The effects of age and marital status would also appear to interact with sex to influence the importance of individual sources of happiness [14]. This is not surprising given that they have been shown to interact in predicting overall levels of happiness or well-being. For example, some research has suggested that the transition to parenthood links to mental well-being, and this differs across the sexes [16,17]. However, other research suggests that the sex gap in the emotional implications of parenthood has dissipated [18]. On a similar note, sex has been found to interact with marital status in predicting levels of happiness [19].

This paper expands on existing research by examining how sex and time frame (last 24 hours vs last 3 months) interact with demographics like age, marital status, and parenthood in predicting sources of happiness. It offers new insights not only by exploring how these factors influence well-being over different time periods but also by considering them in relation to the 5 primary sources of happiness. Mohamed and Mostafa [13] examined the topics of sources of happiness, through natural language processing, and Asai et al [9] identified that statements of happiness can be classified within 7 categories (Table 1). Two of these are endorsed much less frequently than the others (across 24-hour and 3-month time periods; Table 1), but the five most popular sources of happiness are (1) affection, (2) bonding, (3) leisure, (4) achievement, and (5) enjoying the moment [9]. This research focuses on these 5 most common sources of happiness.

These 5 sources shall be focused upon within this paper. The importance of affection aligns with research showing links between romantic relationships and mental health [20]. The importance of achievement concurs with Walsh et al [21] in their conclusion that happiness is associated with career success [21]. Indeed, while much research is correlational, they conclude that happiness actually often precedes career success, in that positive emotions lead to improved outcomes in the workplace [21].

**Table 1:** The categories of happy moments: definitions, examples, and frequency of occurrence.

Category	Definition	Examples	Happiness statements within the 24-hour category (n=49,831), n (%)	Happiness statements within the 3-month category (n=50,704), n (%)
Achievement	With extra effort to achieve a better-than-expected result	Finish work Complete marathon	15,398 (30.9)	18,507 (36.5)
Affection	Meaningful interaction with family, loved ones, and pets	Hug Cuddle Kiss	16,295 (32.7)	17,797 (35.1)
Bonding	Meaningful interaction with friends and colleagues	Have meals with coworker Meet friends	5182 (10.4)	5476 (10.8)
Enjoy the Moment	Being aware or reflecting on present environment	Have a good time Mesmerize	6628 (13.3)	4513 (8.9)
Leisure	An activity done regularly in one’s free time for pleasure	Play games Watch movies Bake cookies	4335 (8.7)	3093 (6.1)
Nature	In the open air and in nature	Garden Beach Sunset Weather	1046 (2.1)	761 (1.5)
Exercise	With intent to exercise or workout	Run Bike Do yoga Lift weights	747 (1.5)	406 (0.8)

### Objectives

This paper uses the HappyDB database to examine whether age category, marital status, and parenthood status predict the likelihood of selecting or not selecting each of the 5 most popular sources of happiness (affection, bonding, leisure, achievement, and enjoying the moment) and whether this differs by sex and time frame. Results will be discussed within a preliminary discussion of how open datasets such as HappyDB may represent a novel method to inform the content design of positive psychology interventions (PPIs), and indeed digital mental health interventions (DMHIs) in general. This domain brings great potential, but the ethical and responsible design of DMHIs brings many considerations and calls for creative approaches [22,23]. Best practice guidelines for the development of DMHIs recommend evidence-based approaches; theory-driven design; tailoring the DMHIs to the population and the context; and consideration of engagement, inclusivity, accessibility, and flexibility [22,24,25]. This represents a significant challenge, as collaboration is needed across many disciplines, and open datasets may have valuable contributions to make.

## Methods

### Materials

There are 100,535 statements within the HappyDB database, with 49,831 statements relating to what made the individual happy in the past 24 hours and 50,704 relating to what made the individual happy within the last 3 months [9]. Table 1 illustrates definitions of each of the 7 categories and examples of the topics discussed within each category. Examples of statements include “My son gave me a big hug in the morning when I woke him up,” “I finally managed to make 40 pushups,” “I had dinner with my husband,” “Morning started with the chirping of birds and the pleasant sun rays,” “The event at work was fun. I loved spending time with my good friends and laughing,” or “I went to the park with the kids. The weather was perfect” [9].

### Ethical Considerations

HappyDB is an open, publicly available dataset that has been used in many other research studies. No personally identifiable information is included in the datasets. Nonetheless, this study received ethical approval from the Filter Committee of the School of Psychology at Ulster University (FCPSYCH2020EE).

### Participants

Sample details are outlined in Table 2.

**Table 2:** Sample details.

Variable			Overall sample (N=100,535), n (%)		24-hour data (n=49,831), n (%)		3-month data (n=50,704), n (%)
**Sex**							
	Male	57,661 (57.4)		28,483 (49.4)		20,937 (49.8)	
	Female	42,036 (41.8)		29,178 (50.6)		21,099 (50.2)	
**Marital status**							
	Married	41,325 (41.2)		20,925 (42.1)		20,400 (40.3)	
	Divorced or separated	4446 (4.4)		2142 (4.3)		2302 (4.6)	
	Widowed	477 (0.5)		228 (0.5)		249 (0.5)	
	Single	54,091 (53.9)		26,441 (53.2)		27,650 (54.6)	
**Parental status**							
	No children	60,911 (60.6)		29,700(59.7)		31,211 (61.6)	
	Children	39,499 (39.3)		20,072 (40.3)		19,427 (38.4)	
**Age group (years)**							
	18-25	24,138 (24.1)		11,960 (24)		12,178 (24.1)	
	26-64	74,932 (74.7)		37,161 (74.7)		37,771 (74.6)	
	65 and older	1264 (1.3)		609 (1.2)		655 (1.8)	

### Analyses

Analyses took the form of binary regression analyses. Based on Asai et al [9] noting that sources of happiness varied across the time periods, the 24-hour data and 3-month data were analyzed separately. Analyses also considered male and female individuals separately. As noted earlier, exercise and nature were the least frequently endorsed sources of happiness (Table 1). Due to the small group sizes associated with these 2 categories, analyses focused on considering group differences within the remaining top-5 sources of happiness. Both sex and parental status were treated as dichotomous variables. Age was considered an ordinal variable, as it has a natural order but unequal intervals. Marital status, being categorical with no inherent order or ranking, was classified as nominal.

There were 20 binary logistic regression analyses in all. The overall dataset was separated into four separate datasets: (1) sources of happiness for female individuals over the previous 24 hours; (2) sources of happiness for male individuals over the previous 24 hours; (3) sources of happiness for female individuals over the previous 3 months; and (4) sources of happiness for male individuals over the previous 3 months. Within each of the 4 datasets, 5 binary logistic regression analyses were conducted. Each one of these five regressions considered one of the sources of happiness as a dependent variable: (1) the presence or absence of affection as a source of happiness; (2) the presence or absence of bonding as a source of happiness; (3) the presence or absence of leisure as a source of happiness; (4) the presence or absence of achievement as a source of happiness; and (5) the presence or absence of enjoying the moment as a source of happiness. The analysis examined whether each of these could be predicted by the demographic factors of age category, marital status, and parenthood status (predictors) and whether this differed by sex and time frame. Basically, there were 5 forms of regression, and each one was done within each of the 4 datasets, giving rise to 20 binary logistic regression analyses. For age as an independent variable, 18-25 years represented the reference category. For marital status as a predictor, married individuals represented the reference category. For parental status as a predictor, those with no children represented the reference category. The assumptions for the analyses were addressed as follows.

- Binary logistic regression assumes that observations are independent. While individuals could provide multiple reports about their sources of happiness, each report was treated as a separate data entry, ensuring no repeated measures
- Multicollinearity among independent variables was examined using variance inflation factor (VIF) values. VIF values below 5 indicate low multicollinearity, while values above 10 suggest a high degree of multicollinearity [26]. In this study, all VIF values were below 2, confirming no multicollinearity issues.
- The linearity of the logit: assumption applies to continuous independent variables. Since all independent variables in this study are categorical, this assumption is not relevant.
- Outlier detection is not applicable here due to the categorical nature of all variables.

## Results

### Affection

Predictors of affection as a source of happiness are outlined in Table 3. The first category (not selecting affection) was the reference category.

Within male individuals, the different age groups did not differ in their likelihood of selecting or not selecting affection as a source of their happiness. Within female individuals, all age groups were significantly more likely than the 18-25 years age group to select affection as a source of their happiness. This was evident within both the 24-hour (*P*<.001) and 3-month (*P*<.01) reports of sources of happiness.

Within both the male and female data, divorced or separated individuals were significantly more likely than married individuals to select affection as a source of their happiness (*P*<.001). However, across both male and female individuals, those who were widowed did not differ from married individuals in their likelihood of selecting affection as a source of happiness. Within male individuals, those who were single did not differ from married individuals in their likelihood of selecting affection as a source of happiness. Within the female individuals, this was the same within the 3-month data. However, within the data where female individuals had reported their sources of happiness within the last 24 hours, single female individuals were significantly (1.38 times; *P*<.05) more likely than married female individuals to select affection as a source of their happiness.

Within both the male and female data, those with children were significantly less likely than those without children to select affection as a source of their happiness (*P*<.001).

**Table 3:** ORs^a^ and significance for logistic regression on choosing “affection” as a source of happiness by demographics and time frame^b^.

			Male, OR (95% CI)					Female, OR (95% CI)		
			24-hour data		3-month data		24-hour data			3-month data
**Age group (years) with “18-25 years” as reference**										
	26-64	1.13 (0.89-1.45)		1.14 (0.91-1.44)		2.13 (1.64-2.75)***			1.43 (1.12-1.83)**	
	65 and older	1.06 (0.84-1.35)		1.17 (0.94-1.46)		1.55 (1.21-1.98)***			1.21 (0.95-1.52)	
**Marital status with “Married” as reference**										
	Divorced or separated	1.54 (1.43-1.66)***		1.48 (1.38-1.59)***		1.49 (1.38-1.61)***			1.34 (1.24-1.45)***	
	Widowed	1.00 (0.85-1.19		1.13 (0.96-1.32)		1.04 (0.91-1.19)			0.98 (0.87-1.11)	
	Single	1.31 (0.71-2.41)		1.32 (0.81-2.13)		1.38 (1.01-1.88)*			1.26 (0.92-1.71)	
**Parenthood with “No children” as reference**										
	Children	0.60 (0.56-0.65)***		0.63 (0.59-0.68)***		0.56 (0.52-0.60)***			0.51 (0.48-0.55)***	

^a^OR: odds ratio.

^b^Reference category: no selection of affection.

*P<.05.

**P<.01.

***P<.001.

### Bonding

Predictors of bonding as a source of happiness are outlined in Table 4. The first category (not selecting bonding) was the reference category.

For age as a predictor of bonding as a source of happiness, 18-25 years represents the reference category. Within male individuals, the different age groups did not differ in their likelihood of selecting or not selecting bonding as a source of their happiness. Within female individuals, the different age groups did not differ in their likelihood of selecting or not selecting bonding as a source of their happiness over the past 3 months. However, when considering what made them happy over the past 24 hours, female individuals aged 26-64 years were significantly more (1.94 times; *P*<.05) more likely than female individuals aged 18-25 years to select bonding as a source of their happiness. Within reports of happiness in the past 24 hours, female individuals aged 65 years old and older did not differ significantly from those aged 18-25 years in their likelihood of selecting bonding as a source of their happiness.

Within both the male and female data, divorced or separated individuals were significantly less likely than married individuals to select bonding as a source of their happiness (*P*<.001). Within male reports of happiness within the past 24 hours, widowed male individuals were significantly less likely than married male individuals to select bonding as a source of their happiness (*P*<.05). However, widowed and married male individuals did not differ in their likelihood of selecting bonding as a source of their happiness over the past 3 months. Widowed and married female individuals did not differ in their likelihood of selecting bonding as a source of their happiness. Across both male and female individuals, those who were single did not differ from married individuals in their likelihood of selecting bonding as a source of happiness.

Male individuals with and without children did not differ in their likelihood of selecting bonding as a source of their happiness over the past 24 hours. However, within 3-month reports of sources of happiness, male individuals with children were much more likely to endorse something within the bonding category in comparison with male individuals with no children (*P*<.001). Within female individuals, those with children were much more likely to endorse something within the bonding category in comparison with those with no children (*P*<.001).

**Table 4:** ORs^a^ and significance for logistic regression on choosing “bonding” as a source of happiness by demographics and time frame^b^.

		Male, OR (95% CI)		Female, OR (95% CI)	
		24-hour data	3-month data	24-hour data	3-month data
**Age group (years) with “18-25 years” as reference**					
	26-64	0.85 (0.58-1.24)	1.03 (0.69-1.55)	1.94 (1.16-3.24)*	1.03 (0.68-1.58)
	65 and older	0.75 (0.52-1.08)	0.98 (0.66-1.46)	1.52 (0.92-2.50)	0.93 (0.62-1.40)
**Marital status with “Married” as reference**					
	Divorced or separated	0.69 (0.62-0.78)***	0.68 (0.61-0.75)***	0.78 (0.69-0.88)***	0.82 (0.72-0.92)***
	Widowed	0.78 (0.61-1.00)*	0.83 (0.65-1.06)	0.96 (0.77-1.18)	1.13 (0.93-1.38)
	Single	0.83(0.32-2.11)	1.10 (0.55-2.24)	1.20 (0.73-1.96)	0.88 (0.51-1.51)
**Parenthood with “No children” as reference**					
	Children	1.07 (0.96-1.20)	1.24 (1.11-1.39)***	1.26 (1.11-1.42)***	1.61 (1.43-1.82)

^a^OR: odds ratio.

^b^Reference category: no selection of affection.

*P<.05.

**P<.01.

***P<.001.

### Leisure

Predictors of leisure as a source of happiness are outlined in Table 5. The first category (not selecting leisure) was the reference category.

**Table 5:** ORs^a^ and significance for logistic regression on choosing “leisure” as a source of happiness by demographics and time frame^b^.

			Male, OR (95% CI)					Female, OR (95% CI)		
			24-hour data		3-month data		24-hour data			3-month data
**Age group (years) with “18-25 years” as reference**										
	26-64	1.31 (0.80-2.15)		0.85 (0.53-1.36)		0.26 (0.17-0.38)***			0.44 (0.25-0.78)**	
	65 and older	1.40 (0.86-2.28)		0.94 (0.59-1.49)		0.44(0.30-0.63)***			0.85(0.49-1.47)	
**Marital status with “Married” as reference**										
	Divorced or separated	1.18 (1.05-1.31)**		1.08 (0.95-1.22)		0.95 (0.83-1.09)			1.27 (1.08-1.49)**	
	Widowed	1.24 (0.97-1.58)		0.94 (0.70-1.27)		0.95 (0.75-1.21)			1.28 (0.98-1.68)	
	Single	3.11 (1.48-6.53)**		0.95 (0.35-2.63)		0.31 (0.13-0.77)**			0.26 (0.06-1.04)	
**Parenthood with “No children” as reference**										
	Children	1.84 (1.63-2.07)		1.56 (1.36-1.78)***		1.87 (1.63-2.15)***			2.35 (2.00-2.75)***	

^a^OR: odds ratio.

^b^Reference category: no selection of affection.

*P<.05.

**P<.01.

***P<.001.

Results show that within the male individuals of the sample, the different age groups did not differ in their likelihood of selecting or not selecting leisure as a source of their happiness. Within the female individuals of the sample, those aged 26-64 years and those aged 65 years and older were both significantly less likely than those aged 18-25 years to select leisure as a source of their happiness (*P*<.001). This was evident in reports of happiness over the past 3 months and the past 24 hours.

In relation to marital status, divorced or separated male individuals were significantly more (1.18 times; *P*<.01) likely than married male individuals to cite leisure as a source of their happiness over the past 24 hours. However, divorced or separated and married male individuals did not differ in their likelihood of citing leisure as a source of their happiness over the past 3 months. The pattern was reversed among female individuals. Specifically, divorced or separated and married female individuals did not differ in their likelihood of citing leisure as a source of their happiness over the past 24 hours. However, divorced or separated female individuals were significantly more (1.27 times; *P*<.01) likely than married female individuals to cite leisure as a source of their happiness over the past 3 months.

Male individuals with and without children did not differ in their likelihood of selecting leisure as a source of their happiness over the past 24 hours. However, within 3-month reports of sources of happiness, male individuals with children were much more likely to endorse something within the leisure category in comparison with male individuals with no children (*P*<.001). Within female individuals, those with children were much more likely to endorse something within the leisure category in comparison with those with no children (*P*<.001).

### Achievement

Predictors of achievement as a source of happiness are outlined in Table 6. The first category (not selecting achievement) was the reference category.

Results show that within the male individuals of the sample, the different age groups did not differ in their likelihood of selecting or not selecting achievement as a source of their happiness. Within the female individuals of the sample, those aged 26-64 years were significantly less likely than those aged 18-25 years to select achievement as a source of their happiness. This was evident in reports of happiness over the past 3 months (*P*<.05) and the past 24 hours (*P*<.001). However, female individuals aged 65 years and older only differed significantly from those aged 18-25 years in terms of them being less likely (0.74 times; *P*<.05) to cite achievement as a source of their happiness over the past 24 hours. The 2 groups did not differ significantly in their likelihood of endorsing achievement as a source of their happiness over the past 3 months.

In relation to marital status, those who were divorced or separated (male and female individuals) were significantly less likely than their counterparts to select achievement as a source of their happiness (*P*<.001). This was evident within 24-hour reports and those of the past 3 months. Within the 24-hour and 3-month reports, widowed individuals did not differ from their married counterparts in the likelihood of selecting achievement as a source of their happiness. Compared to married male individuals, single male individuals were significantly less (0.39 times; *P*<.05) likely to cite achievement as a source of their happiness over the past 24 hours. However, single and married female individuals did not differ significantly in their likelihood of endorsing achievement as a source of their happiness over the past 3 months. Single and married male individuals did not differ significantly in their likelihood of endorsing achievement as a source of their happiness over the past 24 hours or 3 months.

Within male and female individuals, and within the 24-hour reports and the 3-month reports, those with children were significantly more likely than those without children to select achievement as a source of their happiness (*P*<.001).

**Table 6:** ORs^a^ and significance for logistic regression on choosing “achievement” as a source of happiness by demographics and time frame^b^.

		Male, OR (95% CI)		Female, OR (95% CI)	
		24-hour data	3-month data	24-hour data	3-month data
**Age group (years) with “18-25 years” as reference**					
	26-64	0.96 (0.74-1.25)	0.88 (0.70-1.11)	0.64 (0.49-0.84)***	0.73 (0.57-0.94)*
	65 and older	1.02 (0.80-1.32)	0.84 (0.67-1.05)	0.74 (0.58-0.96)*	0.81 (0.63-1.03)
**Marital status with “Married” as reference**					
	Divorced or separated	0.83 (0.77-0.89)***	0.82 (0.77-0.88)***	0.83 (0.76-0.90)***	0.76 (0.70-0.82)***
	Widowed	0.99 (0.84-1.15)	1.08 (0.93-1.25)	1.12 (0.97-1.28)	0.90 (0.80-1.03)
	Single	0.39 (0.18-0.83)*	0.92 (0.57-1.49)	1.08 (0.78-1.50)	0.84 (0.61-1.16)
**Parenthood with “No children” as reference**					
	Children	1.17 (1.09-1.26)***	1.16 (1.08-1.24)***	1.30 (1.20-1.41)***	1.24 (1.15-1.34)***

^a^OR: odds ratio.

^b^Reference category: no selection of affection.

*P<.05.

**P<.01.

***P<.001.

### Enjoy the Moment

Predictors of enjoying the moment as a source of happiness are outlined in Table 7. The first category (not selecting enjoying the moment) was the reference category.

Within male and female individuals, the age categories did not differ in their likelihood of selecting enjoying the moment as a source of their happiness. This was evident within both the 24-hour and 3-month reports of sources of happiness. Within the 24-hour data, divorced or separated male and female individuals were significantly less likely than their counterparts to cite enjoying the moment as a source of their happiness over the past 24 hours (*P*<.001). However, within the 3-month data, divorced or separated male and female individuals did not differ significantly from their counterparts in their likelihood of citing enjoying the moment as a source of their happiness over the past 24 hours. Within male and female individuals, neither the widowed individuals nor single individuals differed from their married counterparts in their likelihood of selecting enjoying the moment as a source of their happiness. This was evident within both the 24-hour and 3-month reports of sources of happiness.

Within 24-hour reports of happiness, those with children (male and female individuals) did not differ from their counterparts in their likelihood of selecting enjoying the moment as a source of their happiness. However, within 3-month reports of happiness, those with children (male individuals: *P*<.01 and female individuals: *P*<.001) were significantly more likely than their counterparts in their likelihood of selecting enjoying the moment as a source of their happiness.

**Table 7:** ORs^a^ and significance for logistic regression on choosing “enjoying the moment” as a source of happiness by demographics and time frame^b^.

			Male, OR (95% CI)				Female, OR (95% CI)		
			24-hour data		3-month data		24-hour data		3-month data
**Age group (years) with “18-25 years” as reference**									
	26-64	1.29 (0.89-1.87)		1.54 (0.95-2.52)		0.92 (0.63-1.34)		141 (0.87-2.27)	
	65 and older	1.26 (0.88-1.82)		1.57 (0.97-2.55)		0.94 (0.65-1.35)		1.13 (0.71-1.80)	
**Marital status with “Married” as reference**									
	Divorced or separated	0.82 (0.75-0.91)***		0.97 (0.87-1.09)		0.79 (0.71-0.89)***		1.00 (0.88-1.14)	
	Widowed	1.02 (0.83-1.26)		0.79 (0.60-1.04)		0.86 (0.71-0.89)		1.17 (0.95-1.46)	
	Single	1.58 (0.78-3.21)		0.46 (0.15-1.47)		0.70 (0.42-1.15)		1.39 (0.83-2.32)	
**Parenthood with “No children” as reference**									
	Children	0.97 (0.88-1.07)		1.20 (1.07-1.35)**		1.07 (0.95-1.19)		1.31 (1.15-1.49)***	

^a^OR: odds ratio.

^b^Reference category: no selection of affection.

*P<.05.

**P<.01.

***P<.001.

## Discussion

### Principal Findings

This paper set out to (1) present an analysis of how sex may interact with age, marital status, and parental status to predict individual differences in sources of happiness; and (2) present a preliminary discussion of how open datasets may contribute to the process of designing health-related technology innovations. In terms of a brief summary of findings, sex interacted with age, marital status, and parental status in predicting sources of happiness, with some patterns varying across time periods.

Earlier research has suggested that sex interacts with other demographic factors to predict individual differences in sources of happiness [14]. Results partially supported this suggestion. First, sex and age interacted in predicting sources of happiness. Clear trends were evident in the data for male individuals. Male individuals across the 3 age categories did not differ in their likelihood of reporting affection, bonding, leisure, achievement, or enjoying the moment as sources of happiness. Trends were not as consistent within the female data, with the time period under consideration also exerting significant effects on the likelihood of affection, bonding, leisure, and achievement being reported as a source of happiness. The exact direction of effects is described in the *Results* section. The only exception was in relation to enjoying the moment as a source of happiness wherein, like the male group, the different female age groups did not differ in their likelihood of reporting enjoying the moment as a source of happiness. This was regardless of the time period under consideration. Results can therefore be said to only partially agree with a previous review by Buijs et al [27], which concluded that chronological age did not relate to affection, status, and other behavioral factors as sources of happiness.

Results partially tied in with earlier conclusions that sex interacts with marital status in predicting levels of happiness [19]. Across the board, differences in sources of happiness were predominantly between married individuals and those who are divorced or separated. However, these differences were the same for both sexes. Married, single, and widowed individuals were all largely similar in their likelihood of selecting each of the categories as a source of their happiness. However, there were some anomalies and sex appeared to be important in these anomalies. For example, in comparison to married male individuals, single male individuals were less likely to report achievement as a source of their happiness in the previous 24 hours. On the contrary, married and single female individuals did not differ in their likelihood of reporting achievement as a source of their happiness in the previous 24 hours. Compared to married male individuals, single male individuals were significantly more likely to report leisure as a source of their happiness in the past 24 hours. Conversely, in comparison with married female individuals, single female individuals were significantly less likely to report leisure as a source of happiness in the previous 24 hours. Such patterns were not evident in the 3-month data. It must be acknowledged that the current study only looks at marital status, whereas other factors such as commitment are also important [20]. Understanding how marital status ties to happiness is important, as there is a bidirectional relationship between relationships and mental health, which is particularly strong when relationship status is used as a predictor of health [20].

Parental status and sex also appeared to interact in predicting some, but not all, sources of happiness. Compared to those who were not parents, those who were parents were significantly less likely to consider affection as a source of their happiness and significantly more likely to consider achievement as a source of their happiness. However, differences were apparent within both the male and female data, and across both the 24-hour and 3-month time periods. Compared to male individuals who were not parents, those male individuals who were parents were significantly more likely to consider enjoying the moment as a source of their happiness over the past 3 months, but not the past 24 hours. This exact same pattern was evident in the female data. However, as can be seen from the results, the sexes did differ in how they reported bonding and leisure as sources of happiness. Some past research has suggested sex differences in how the transition to parenthood links to mental well-being [16,17], and some have suggested that the sex gap in the emotional implications of parenthood has dissipated [18]. The current findings are consistent with the inconclusive nature of past research, and the recognized need to consider the complexity of how and why parents experience happiness [28].

### Limitations

Limitations of course must be considered. As the HappyDB data were gathered via crowdsourcing, the representativeness of the sample is unknown. Obviously, data collection was dependent upon access to technology. Many other demographic factors link to happiness, for example, health and socioeconomic status. Sample details are not available for factors such as socioeconomic status or health conditions, but the US background and the limited representation of older participants are clear limitations in terms of the cultural and age group representativeness of this analysis. Open datasets often lack details on psychological factors, such as personality traits, that influence individual differences in happiness [29]. To maximize their use in developing DMHIs, researchers must balance the breadth and depth of data. While large sample sizes offer generalizable insights and scalability, incorporating psychometric measures (eg, personality or emotional well-being scales), and providing richer, more personalized data. A balanced approach that integrates demographic diversity with targeted, detailed subsamples would ensure both robust and effective DMHIs. Similarly, within the Positive Emotions, Engagement, Relationships, Meaning, Accomplishment, and Health model, happiness is just 1 example of positive emotion, and many other components link to well-being (eg, engagement, relationships, meaning, accomplishments, and health). This paper analyzes each source of happiness independently. However, exploring correlations between sources within demographics could offer deeper insights. For instance, responses about affection, bonding, leisure, achievement, or enjoying the moment might reflect the same “event” over different time frames, with patterns varying by demographic. It would be interesting to examine how many individuals fall into the same happiness category for both the 24-hour and 3-month statements by demographic group, and whether certain demographics are more likely to report consistent happiness across time frames.

### Conclusions

There currently are no best practice guidelines to support developers and designers in gathering and defining the stakeholder-driven or humanity-centric requirements, use cases, and goals for DMHIs in select domains. The ethical and responsible design of DMHIs should be evidence-based approaches; use theory-driven design; be tailored to the population and the context; and be considerate of engagement, inclusivity, accessibility, and flexibility, but this represents a significant challenge [22,24,25]. However, the identification of universal and domain-specific needs is also central to user engagement and retention. Coproduction would typically include interviews, focus groups, literature reviews, workshops, panel discussions, and clinical expertise [23]. Of course, such processes are very time, and as such resource intensive [23].

Within their systematic review, Brotherdale et al [23] note the need for creative methods to be adopted in the coproduction of digital mental health intervention, and the use of open datasets may be a fruitful approach in this endeavor. Currently, little attention has been paid to open datasets as a creative approach to requirements gathering and informing content development of PPIs, and indeed DMHIs in general. The HappyDB analysis can be presented as a sample application.

PPIs can be tailored to address diverse challenges and promote well-being, as well as prevent mental ill health by promoting protective factors, enhancing resilience, and fostering well-being [30]. PPIs often involve suggested activities to enhance happiness or well-being [9]. PPIs generally focus on one of five categories: (1) savoring experiences and sensations; (2) cultivating (and sometimes expressing) gratitude; (3) engaging in kind acts; (4) promoting positive relationship processes; and (5) pursuing hope and meaning [31]. Their mode of operation is typically either diaries for self-reflection or applications that make suggestions based on responses to set questions [9]. Some examples relevant to this discussion might be virtual reality interventions that seek to promote happiness by immersing users in positive environments or guided meditations; strength-based platforms that help users to identify and use their personal strengths; positive psychology games that are designed to promote positive emotions; or gratitude apps where users can keep a digital gratitude journal, but there would also be reminders and prompts to help them focus on positive aspects of their lives.

User engagement difficulties across the board represent a major challenge to PPIs, and indeed DMHIs in general, achieving their maximum potential benefits [32]. Designing content that can be personalized based on user data, preferences, and progress to increase engagement and effectiveness represents a major challenge for design developers, with collaboration across several disciplines required [32]. However, as can be seen earlier, notwithstanding the recognized limitations, open datasets such as the HappyDB provide great insight into how demographics link to differences in sources of happiness. Certain PPIs or suggested activities may have increased meaning for certain subgroups, and open datasets may provide a starting point for understanding what these might be. Research on the science of happiness is also relevant to the concept of intrinsic capacity for healthy aging and holistic well-being. This concept remains undervalidated, and a deeper understanding of happiness could help validate and practically apply it in mental health interventions [33]. Open datasets may also come in various formats. For example, there are many sources that outline counseling dialogues, and how these are rated by users. Through the use of machine learning, these may give insights into useful content generation for DMHIs.

When discussing DMHI content design, it is important to consider the lack of context in interpreting the significance of insights derived from open-source data. This is a key limitation in health technology design. Evaluating user satisfaction and engagement is essential to determine whether a version created using open data is received more positively than one developed through other approaches. Metrics such as usability, satisfaction, and effectiveness should be compared to assess the value of incorporating open data in DMHI development.

Many platforms host open datasets, with the Open Science Framework, Mendeley Data, PsychOpen Community, Archive, and Metadata for Open Science, or Kaggle representing examples. Nonetheless, supporting researchers in providing findable, accessible, interoperable, and reusable data poses significant challenges [8]. For example, what might a framework to describe the nature of the data that are typically gathered across different domains look like? What would the best methods be for structuring data (and metadata) that are human and machine readable but also useful for designers and analysts and for interpretability across different channels? How would datasets be described in terms of purposes, goals, and communication patterns, and what might a structured representational repository look like?

Huston [7] discusses the challenges associated with open data in terms of three categories: (1) making the technological shift; (2) social, cultural, legal, and ethical issues; and (3) avoiding the pitfalls. Nonetheless, a discussion of the extent to which our understanding of data and interactions from nondigital settings may inform requirement gathering for DMHIs is warranted. While the challenges associated with open data are substantial and should not be underestimated, the potential benefits of open data are very exciting [7]. They may play a role in the development of content for digital PPIs that are engaging, effective, and grounded in scientific research.

## Abbreviations

DMHI: digital mental health intervention
PPI: positive psychology intervention
VIF: variance inflation factor

## Data Availability

The HappyDB is a dataset containing over 100,000 happy statements [9] that is freely available on the web [10]. The data set analyzed during this study is available on the Megagon Labs website [34].
